# Enhancing Cubes with Models to Describe Multidimensional Data

**DOI:** 10.1007/s10796-021-10147-3

**Published:** 2021-06-11

**Authors:** Matteo Francia, Patrick Marcel, Verónika Peralta, Stefano Rizzi

**Affiliations:** 1 DISI, University of Bologna, Bologna, Italy; 2 LIFAT, University of Tours, Tours, France

**Keywords:** OLAP, Models, Multidimensional data, Data exploration

## Abstract

The Intentional Analytics Model (IAM) has been recently envisioned as a new paradigm to couple OLAP and analytics. It relies on two basic ideas: (i) letting the user explore data by expressing her analysis intentions rather than the data she needs, and (ii) returning enhanced cubes, i.e., multidimensional data annotated with knowledge insights in the form of interesting model components (e.g., clusters). In this paper we contribute to give a proof-of-concept for the IAM vision by delivering an end-to-end implementation of describe, one of the five intention operators introduced by IAM. Among the research challenges left open in IAM, those we address are (i) automatically tuning the size of models (e.g., the number of clusters), (ii) devising a measure to estimate the interestingness of model components, (iii) selecting the most effective chart or graph for visualizing each enhanced cube depending on its features, and (iv) devising a visual metaphor to display enhanced cubes and interact with them. We assess the validity of our approach in terms of user effort for formulating intentions, effectiveness, efficiency, and scalability.

## Introduction

Data warehousing and OLAP (On-Line Analytical Processing) have been progressively gaining a leading role in enabling business analyses over enterprise data since the early 90’s. During these thirty years, the underlying technologies have evolved from the early relational implementations (still widely adopted in corporate environments), to the new architectures solicited by Business Intelligence 2.0 scenarios, and up to the challenges posed by the integration with big data settings. However, recently, it has become more and more evident that the OLAP paradigm alone is no longer sufficient to keep the pace with the increasing needs of new-generation decision makers. Indeed, the enormous success of machine learning techniques has consistently shifted the interest of corporate users towards more sophisticated analytical applications (Popovic et al. 2018; Schuff et al. 2018). In addition, recent research envisions cross-cutting data management, analytics, and artificial intelligence in various sectors, such as applied data science (Chiusano et al. 2021), behavioral research (Motiwalla et al. 2019) and social impact (Gupta et al. 2018).

**Fig. 1:**
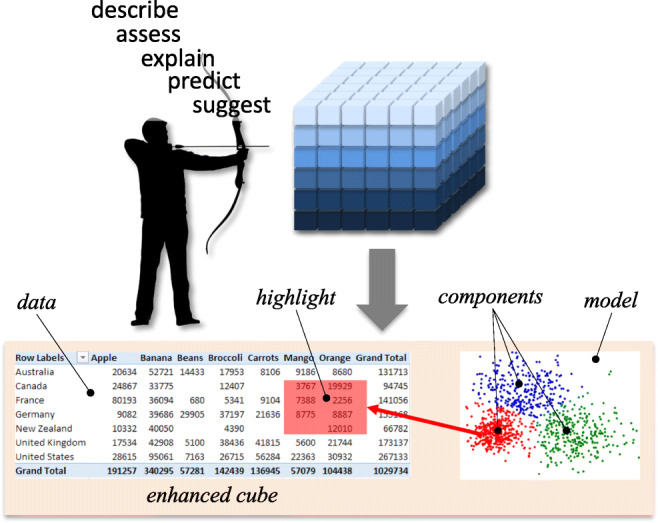
The IAM approach: the user expresses an intention and receives in return an enhanced cube

In this direction, the *Intentional Analytics Model* (IAM) has been envisioned as a way to tightly couple OLAP and analytics (Vassiliadis et al. 2019). As sketched in Fig. 1, the IAM approach relies on two major cornerstones: (i) the user explores the data space by expressing her analysis *intentions* rather than by explicitly stating what data she needs, and (ii) in return she receives both multidimensional data and knowledge insights in the form of annotations of interesting subsets of data.

As to (i), five intention operators are proposed, namely, describe (describes one or more cube measures, possibly focused on one or more level members), assess (judges one or more cube measures with reference to some baseline), explain (reveals some hidden information in the data the user is observing, for instance in the form of a correlation between two measures), predict (shows data not in the original cubes, derived for instance with regression), and suggest (shows data similar to those the current user, or similar users, have been interested in). As to (ii), first-class citizens of the IAM are *enhanced cubes*, defined as multidimensional cubes coupled with *highlights*, i.e., sets of cube cells associated with interesting components of *models* automatically extracted from cubes. Each operator is applied to an enhanced cube and returns a new enhanced cube. To assess the interestingness of model components, a measure based on their significance — expressed in terms of how novel, peculiar, and surprising they are expected to be to the user — is used. Noticeably, having different models automatically computed and evaluated in terms of their interestingness relieves the user from the time-wasting effort of trying different possibilities.

### *Example 1*

Let a SALES cube be given, and let the user’s intention be

$$ \begin{array}{@{}rcl@{}} &&\textsf{with SALES describe quantity}\\ &&  \textsf{for month = '1997-04' by type}\\ &&  \textsf{using outliers} \end{array} $$

**Fig. 2:**
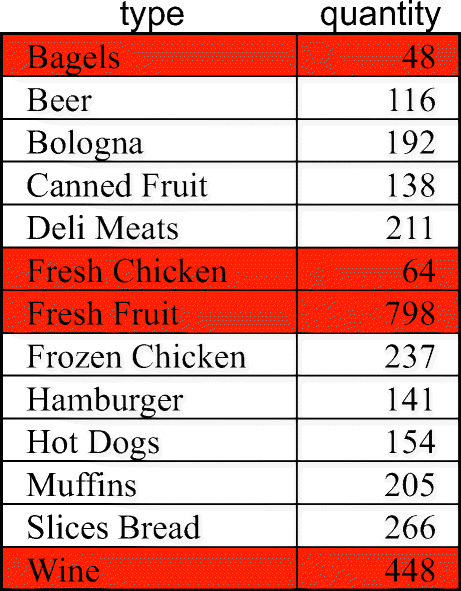
The enhanced cube resulting from the intention in Example 1; the highlight is in red

Firstly, the subset of cells for April 1997 are selected from the SALES cube, aggregated by product type, and projected on measure quantity (in OLAP terms, a slice-and-dice and a roll-up operator are applied). Then, the outliers are found in these cells based on the values of quantity. Finally, a measure of interestingness is computed for the two components obtained (the outlier cells, and the non-outlier ones), and the cells belonging to the component with maximum interestingness (in this case, outlier cells) are highlighted in the results shown to the user (see Fig. 2).

The IAM vision aims at facilitating exploratory analysis by redefining queries and answers, and by providing the user with a declarative language that enables her to specify her analytical intentions (Vassiliadis et al. 2019). Such a paradigm shift necessarily includes a degree of automation, and a balance is to be sought between the implementation of the analytical intentions and the freedom left to the user to specify it. This raises a number of research challenges, e.g., (i) investigate if there are any other intention operators that should be considered besides the basic ones proposed, and how different operators can be combined; (ii) find techniques for automatically tuning the algorithms that create enhanced cubes by computing models; (iii) devise a measure to estimate the interestingness of model components; (iv) enrich the IAM framework with an approach to select the most effective chart or graph for visualizing each cube depending on its features such as number of dimensions, size, etc.; and (v) devise a visual metaphor for displaying enhanced cubes and interacting with them.

In the direction of providing a proof-of-concept for the IAM vision, the potentiality of the assess operator has been recently investigated by proposing a syntax, a semantics, and a basic optimization strategy (Francia et al. 2021). The goal of this paper is to take one step forward in the same direction by delivering an end-to-end implementation of the describe operator. Specifically, we address challenge (ii) by experimenting two techniques to automatically set the number of model components, and challenge (iii) by proposing and validating a new interestingness measure for model components. Notably, this measure is consistent with the multi-facets interestingness scheme introduced by Marcel et al. (2019). The present work gives a precise and motivated definition for both the facets used and the way they are aggregated to form a global score. We also address challenges (iv) and (v), by proposing a visualization that couples text-based representations and selected graphical representations with a component-driven interaction paradigm. In this way, the user will save the time required to try different visualizations; besides, by automatically selecting the most suitable charts based on the features of each cube, we discourage the user from adopting inappropriate visualizations which might lead her to wrong interpretations of data.

This paper significantly extends our previous work (Chédin et al. 2020) in different ways:

- Cube schemata are defined in more general terms, allowing branches in hierarchies rather than only allowing linear hierarchies.
- A new definition of interestingness is given based on three different facets of model components: surprise, novelty, and peculiarity.
- The computation of interestingness is generalized to cover situations where an intention changes both the group-by set and the selection predicate of the previous intention, and when there is no roll-up/drill-down relationship between the two group-by sets.
- The syntax of the describe operator has been extended.
- The visualization of enhanced cubes uses two more chart types to give users a more comprehensive and flexible description of data.
- The approach is evaluated through a comprehensive set of tests not only in terms of efficiency, but also of scalability, effectiveness, and formulation complexity.

The paper outline is as follows. After introducing a formalism to manipulate cubes and queries in Section 2, in Section 3 we introduce models, components, and enhanced cubes, and in Section 4 we define an interestingness measure. Then, in Section 5 we show how an intention is transformed into an execution plan, in Section 6 we discuss how to automatically set the model size, i.e., its number of components, and in Section 7 we explain how enhanced cubes are visualized. Section 8 shows the results of the experimental tests we performed to evaluate the approach. Finally, in Section 9 we discuss the related literature, while in Section 10 we draw the conclusion.

## Formalities

In this section we introduce the formal notations we will use in the paper to manipulate cubes. We start by defining cube schemata; note that the definitions we give support to hierarchies with branches and diamonds.

### **Definition 1** (Hierarchy and Cube Schema)

A *hierarchy* is a triple *h* = (*L*_*h*_,≽_*h*_,≥_*h*_) where:

(i) (*L*_*h*_,≽_*h*_) is a *roll-up* lattice^1^ of categorical *levels*;
(ii) each level *l* ∈ *L*_*h*_ is coupled with a *domain* *Dom*(*l*) including a set of *members*; and
(iii) (*L*,≥_*h*_), where $L = \bigcup _{l \in L_{h}} Dom(l)$, is a *part-of* partial order.

The top level of ≽_*h*_ is called *dimension*. The bottom level, denoted *ALL*_*h*_, has a single member ALL_*h*_. The part-of partial order is such that, for each couple of levels *l* and *l*^′^ such that $l \succeq _{h} l^{\prime }$ and for each member *u* ∈ *Dom*(*l*), there is exactly one member $u^{\prime } \in Dom(l^{\prime })$ such that $u \geq _{h} u^{\prime }$. A *cube schema* is a couple $\mathcal {C}=(H, M)$ where:

(i) *H* is a set of hierarchies;
(ii) *M* is a set of numerical measures, with each measure *m* ∈ *M* coupled with one aggregation operator *op*(*m*) ∈{sum, avg,…}.

### *Example 2*

For our working example it is SALES = (*H*, *M*) where

$$ \begin{array}{@{}rcl@{}} H&=&\{h_{\textsf{Date}},h_{\textsf{Customer}},h_{\textsf{Product}},h_{\textsf{Store}}\}\\ M&=&\{\textsf{quantity}, \textsf{storeSales}, \textsf{storeCost}\} \\ op(\textsf{quantity})&=&op(\textsf{storeSales})=op(\textsf{storeCost})=\textup{\texttt{sum}} \end{array} $$

**Fig. 3:**
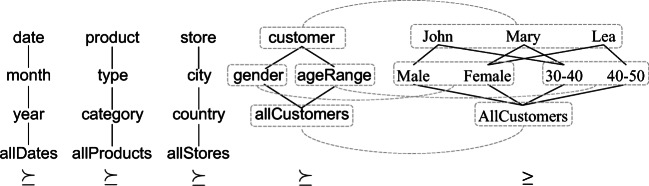
Roll-up lattices (left) and an excerpt of the part-of partial order (right) for the SALES cube in Example 2

The roll-up lattices of the hierarchies in *H* are shown in Fig. 3 together with an excerpt of the part-of partial order of the customer hierarchy. Intuitively, having customer ≽_Customer_gender means that customers can be grouped based on their gender, and having Mary ≥_Customer_Female means that Mary belongs to the group of females.

Aggregation is the basic mechanism to query cubes, and it is captured by the following definition of group-by set.

### **Definition 2** (Group-by Set and Coordinate)

Given cube schema $\mathcal {C}=(H, M)$, a *group-by set* *G* of $\mathcal {C}$ is a set of levels, at least one from each hierarchy of *H*, such that for each couple of levels $l,l^{\prime } \in G$, $l,l^{\prime } \in L_{h}$, we have $l \not \succeq _{h} l^{\prime }$ and $l^{\prime } \not \succeq _{h} l$. The lattice induced on the set of all group-by sets of $\mathcal {C}$ by the roll-up lattices of the hierarchies in *H*, is denoted with ≽_*H*_ and called *multidimensional lattice*. A *coordinate* of a group-by set *G* is a tuple of members, one for each level of *G*. The partial order induced on the set of all coordinates of $\mathcal {C}$ by the part-of partial orders of the members in *H*, is denoted with ≥.

Intuitively, given two group-by sets *G* and $G^{\prime }$, if $G \succeq _{H} G^{\prime }$ (*G* *roll-ups* to $G^{\prime }$) then the coordinates of *G* can be grouped by $G^{\prime }$; given two specific coordinates of *G* and $G^{\prime }$, namely, *γ* and $\gamma ^{\prime }$, if $\gamma \geq \gamma ^{\prime }$ (*γ* *is part of* $\gamma ^{\prime }$) then *γ* belongs to the group defined by $\gamma ^{\prime }$.

To support the definition of interestingness in Section 4, we need to introduce a further notation to establish a mapping between coordinates of different group-by sets. Given two members *u* and $u^{\prime }$ of levels *l* and $l^{\prime }$ both belonging to the same hierarchy *h*, we will write $u \lesseqgtr u^{\prime }$ when either (i) $l=l^{\prime }$ and $u=u^{\prime }$, or (ii) $l \succeq _{h} l^{\prime }$ and $u \geq _{h} u^{\prime }$, or (iii) $l^{\prime } \succeq _{h} l$ and $u^{\prime } \geq _{h} u$. Intuitively, this means that there is a directed path in the part-of partial order connecting the two members, so one of them is an ancestor of the other. Given two coordinates *γ* and $\gamma ^{\prime }$ of two group-by sets *G* and $G^{\prime }$, we will write $\gamma \lesseqgtr \gamma ^{\prime }$ when $\forall u \in \gamma , \exists u^{\prime } \in \gamma ^{\prime } : u \lesseqgtr u^{\prime }$. Note that $\gamma \lesseqgtr \gamma ^{\prime } \Leftrightarrow \gamma ^{\prime } \lesseqgtr \gamma $.

### *Example 3*

**Fig. 4:**
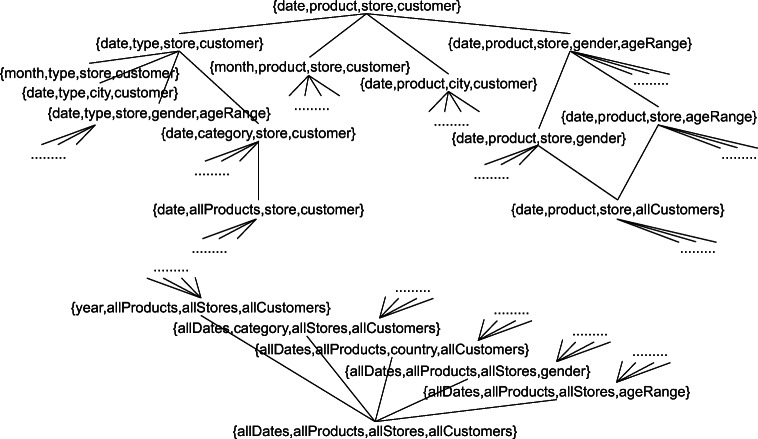
An excerpt of the multidimensional lattice for the SALES cube

Three group-by sets of SALES are

$$ \begin{array}{@{}rcl@{}} G_{1}&=&\{\textsf{date},\textsf{allCustomers},\textsf{type},\textsf{country}\}\\ G_{2}&=&\{\textsf{month},\textsf{allCustomers},\textsf{category},\textsf{allStores}\}\\ G_{3}&=&\{\textsf{year},\textsf{gender},{\textsf{ageRange},}\textsf{category},\textsf{country}\} \end{array} $$

where *G*_1_ ≽_*H*_*G*_2_ while *G*_3_ is incomparable with both *G*_1_ and *G*_2_ (i.e., the coordinates of *G*_3_ cannot be grouped by *G*_1_ and *G*_2_, and vice versa). *G*_1_ aggregates sales by date, product type, and store country, *G*_2_ by month and category, *G*_3_ by year, gender, age range, category, and country. A small excerpt of the multidimensional lattice is shown in Fig. 4. Example of coordinates of the three group-by sets are, respectively,

$$ \begin{array}{@{}rcl@{}} \gamma_{1}&=&\langle \textup{1997-04-15},\textup{AllCustomers},\textup{Fresh Fruit},\textup{Italy}\rangle\\ \gamma_{2}&=&\langle \textup{1997-04},\textup{AllCustomers},\textup{Fruit},\textup{AllStores}\rangle\\ \gamma_{3}&=&\langle \textup{1997},\textup{Female},{\textsf{[30-39]},}\textup{Fruit},\textup{France}\rangle \end{array} $$

where *γ*_1_ ≥ *γ*_2_ (meaning that *γ*_1_ is part of *γ*_2_), while *γ*_3_ is incomparable in the part-of partial order with both *γ*_1_ and *γ*_2_ (meaning that none of them is part of the other). We also have $\gamma _{1} \lesseqgtr \gamma _{2}$ (because, for all levels, members are either the same — as for allCustomers — or one is an ancestor of the other — as 1997-04 for 1997-04-15), $\gamma _{1} \not \lesseqgtr \gamma _{3}$ (because Italy is incomparable with France, i.e. no one is an ancestor of the other), and $\gamma _{2} \lesseqgtr \gamma _{3}$.

The instances of a cube schema are called cubes and are defined as follows:

### **Definition 3** (Cube)

A *cube* over $\mathcal {C}$ is a tuple *C* = (*G*_*C*_, *M*_*C*_, *ω*_*C*_) where:

(i) *G*_*C*_ is a group-by set of $\mathcal {C}$;
(ii) $M_{C} \subseteq M$;
(iii) *ω*_*C*_ is a partial function that maps some coordinates of *G*_*C*_ to a numerical value for each measure *m* ∈ *M*_*C*_.

The function is partial since cubes are normally *sparse*: not all possible business events actually occur, and a coordinate participates in the function only if the event it describes took place. Each coordinate *γ* that participates in *ω*_0_, with its associated tuple *t* of measure values, is called a *cell* of *C* and denoted 〈*γ*, *t*〉. With a slight abuse of notation, we will also consider a cube as the set of the coordinates corresponding to its cells, so we will write *γ* ∈ *C* to state that 〈*γ*, *t*〉 is a cell of *C*.

A cube whose group-by set *G*_*C*_ includes all and only the dimensions of the hierarchies in *H* and such that *M*_*C*_ = *M*, is called a *base cube*, the others are called *derived cubes*. In OLAP terms, a derived cube is the result of either a roll-up, a slice-and-dice, or a projection made over a base cube; this is formalized as follows.

### **Definition 4** (Cube Query)

A *query* over cube schema $\mathcal {C}$ is a triple *q* = (*G*_*q*_, *P*_*q*_, *M*_*q*_) where:

(i) *G*_*q*_ is a group-by set of *H*;
(ii) *P*_*q*_ is a (possibly empty) set of selection predicates, each expressed over one level of *H* using either a comparison operators (=, ≥, etc.) or the set inclusion operator (e.g., country in Italy, France);
(iii) $M_{q} \subseteq M$.

Let *C*_0_ be a base cube over $\mathcal {C}$. The result of applying *q* to *C*_0_ is a derived cube *C* = *q*(*C*_0_) such that (i) *G*_*C*_ = *G*_*q*_, (ii) *M*_*C*_ = *M*_*q*_, and (iii) *ω*_*C*_ assigns to each coordinate *γ* ∈ *C* satisfying the conjunction of the predicates in *P*_*q*_ and to each measure *m* ∈ *M*_*C*_ the value computed by applying *op*(*m*) to the values of *m* for all the coordinates $\gamma ^{\prime }$ of *C*_0_ such that $\gamma ^{\prime } \geq \gamma $.

### *Example 4*

The cube query over SALES used in Example 1 is *q* = (*G*_*q*_, *P*_*q*_, *M*_*q*_) where *G*_*q*_ = {allDates,allCustomers,type,allStores}, *P*_*q*_ = {month = ’1997-04’}, and *M*_*q*_ = {quantity}. A cell of the resulting cube *q*(SALES_0_) (where SALES_0_ is the base cube) is 〈AllDates,AllCustomers,Canned Fruit,AllStores〉 with associated value 138 for quantity.

## Enhancing Cubes with Models

Models are concise, information-rich knowledge artifacts (Terrovitis et al. 2007) that represent relationships hiding in the cube cells. The possible models range from simple functions and measure correlations to more elaborate techniques such as decision trees, clusterings, etc. A model is bound to (i.e., is computed over the levels/measures of) one cube, and is made of a set of components (e.g., a clustering model is made of a set of clusters). In the IAM, a relevant role is taken by data-to-model mappings. Indeed, a model partitions the cube on which it is computed into two or more subsets of cells, one for each component (e.g., the subsets of cells belonging to each cluster).

### **Definition 5** (Model and Component)

A *model* is a tuple ${\mathscr{M}}=(t,alg,C,$ *In*, *Out*, *μ*) where:

(i) *t* is the model type;
(ii) *alg* is the algorithm used to compute *Out*;
(iii) *C* is the cube to which ${\mathscr{M}}$ is bound;
(iv) *In* is the tuple of levels/measures of $\mathcal {C}$ and parameter values supplied to *alg* to compute ${\mathscr{M}}$;
(v) *Out* is the set of components that make up ${\mathscr{M}}$;
(vi) *μ* is a function mapping each coordinate of *C* to one component of *Out*.

Each model *component* is a tuple of a component identifier plus a variable number of properties that describe that component.

In the scope of this work, it is *t* ∈{top-k,bottom-k, skyline,outliers,clustering}. The components for these model types are as follows:

1. For *t* = top-k, there are two components: one for top-k cells, one for the others (similarly for bottom-k). Each component is described by the average z-score of its cells.
2. For *t* = skyline, there are two components: one for the cells in the skyline, one for the others. Each component is described by the average z-score of its cells. To compute the skyline, we resort to the algorithm proposed by Chomicki et al. (2003).
3. For *t* = outliers, there are two components: one for outlier cells, one for the others. Each component is described by its outlierness.^2^ To compute outliers, we adopt the *isolation forest* algorithm (Liu et al. 2008).
4. For *t* = clustering, there is one component for each cluster. Each component is described by the centroid of the corresponding cluster. To compute clustering we resort to the well-known k-means algorithm.

The model types listed above are suggested in the original proposition of the IAM as those that best meet the goal of *describing* a cube (Vassiliadis et al. 2019). Other effective model types are not taken into account here because they were considered to better meet the goals of other intentional operators (e.g., correlation and regression are used to *explain*, time-series decomposition and auto-regression to *predict*). We also note that the properties mentioned for each model type are not meant to be exhaustive.

### *Example 5*

A possible model over the derived cube *q*(SALES_0_) in Example 4 is characterized by

$$ \begin{array}{@{}rcl@{}} &&t = \text{clustering}, alg =\text{K-Means}, C =q(\textsf{SALES}_{0}),\\ &&In =\langle \textsf{quantity},n=3, rndSeed=0 \rangle, Out =\{c1, c2, c3 \}, \\ &&\mu(\langle \text{AllDates},\text{AllCustomers},\text{Bagels},\text{AllStores} \rangle)=c1;\\ &&\mu(\langle \text{AllDates},\text{AllCustomers},\text{Beer},\text{AllStores} \rangle)= c1;\\ &&\mu(\langle \text{AllDates},\text{AllCustomers},\text{Bologna},\text{AllStores} \rangle) =c2;\\ &&\mu(\langle \text{AllDates},\text{AllCustomers},\text{Canned Fruit},\text{AllStores} \rangle)=c2;\\ &&\mu(\langle \text{AllDates},\text{AllCustomers},\text{Deli Meats},\text{AllStores} \rangle) =c2;\\ &&\mu(\langle \text{AllDates},\text{AllCustomers},\text{Fresh Chicken},\text{AllStores} \rangle) =c1;\\ &&\mu(\langle \text{AllDates},\text{AllCustomers},\text{Fresh Fruit},\text{AllStores} \rangle)=c3;\\ &&\mu(\langle \text{AllDates},\text{AllCustomers},\text{Frozen Chicken},\text{AllStores} \rangle) =c2;\\ &&\mu(\langle \text{AllDates},\text{AllCustomers},\text{Hamburger},\text{AllStores} \rangle) =c2;\\ && \ldots \end{array} $$

where *n* is the desired number of clusters and *rndSeed* is the seed to be used by the k-means algorithm to randomly generate the 3 seed clusters. Component *c*1 is characterized by property *centroid* with value 76.

As the last step in the IAM approach, cube *C* is enhanced by associating it with a set of models bound to *C* and with a *highlight*, i.e., with the subset of cells corresponding to the most interesting component of the model; these cells are determined via function *μ*.

### **Definition 6**

An *enhanced cube* *E* is a triple of a cube *C*, a set of models $\{{\mathscr{M}}_{1}, \ldots , {\mathscr{M}}_{r}\}$ bound to *C*, and a highlight

$$ c_{high} = argmax_{\{c \in \bigcup_{i=1}^{r} Out_{i}\}}(interest(c)) $$

How to estimate the interestingness of component *c*, *interest*(*c*), is the subject of next section.

## Estimating the Interestingness of Components

The basic idea of the IAM is that the user will work in sessions, similarly to the OLAP paradigm. Thus, starting from a base cube, the user will write a sequence of intentions; each intention, as explained in Section 5, will determine a cube query which will be applied to *C*_0_ to obtain a derived cube. Now let *C*_0_ be a base cube over schema $\mathcal {C}$, *C* be the cube obtained by the current intention, ${\mathscr{M}}=(t,alg,C,In,Out, \mu )$ be a model bound to *C*, and *c* ∈ *Out* be one of the components of ${\mathscr{M}}$.

The measure proposed by Chédin et al. (2020) to assess the interestingness of component *c* is based on the idea of *prior belief* (Bie 2013): specifically, it defines the interestingness of *c* as the difference of belief for corresponding cells in the cube before and after the application of the intention. In this work we develop a more sophisticated model, based on three facets of interestingness identified by Marcel et al. (2019), namely:^3^

- The *novelty* of *c*, which measures its interestingness with respect to the history of the user with *C*_0_ . Intuitively, a component has more novelty if it concerns a larger number of previously-unseen cells.
- The *peculiarity* of *c*, which measures its interestingness with respect to the cells in the cube $C^{\prime }$ obtained by the last intention the user has formulated with *C*_0_. Concretely, we compare the cells belonging to *c* to some related cells in $C^{\prime }$, and we measure to what extent measure values deviate. A component is more peculiar if such difference is higher.
- The *surprise* of *c*, which measures its interestingness with respect to the user’s previous beliefs about *C*_0_. Intuitively, user’s belief are related with what she learned from previous cubes. Then, a component is more surprising if it includes cells that have not been seen frequently.

Therefore, for each component, we give three scores, one for each interestingness facet. We then define the global interestingness as a linear combination of the three facets. Choosing the weights of each facet enables the user to craft their own interestingness score. For instance, in some typical exploratory OLAP scenario, frequently-seen components may still be seen as interesting by the user, who should then switch off novelty and surprise.

### **Definition 7** (Interestingness)

Let *c* be a component of model ${\mathscr{M}}$. The *interestingness* of *c* is defined as

$$ interest(c) = \alpha_{nov} nov(c) + \alpha_{pec} pec(c) + \alpha_{sur} sur(c) $$

where *nov*(*c*), *pec*(*c*), and *sur*(*c*) denote, respectively, the novelty, peculiarity, and surprise of *c*, and the *α*’s are normalized weights.

### Novelty

To define this score, we assume that the system keeps track of the user’s history with *C*_0_ through the set *V* of all the cubes that the user has computed during her current session on *C*_0_.

#### **Definition 8** (Novelty)

Let *c* be a component of model ${\mathscr{M}}$. The *novelty* of *c* is defined as

$$nov(c)=avg_{\gamma \in \mu^{-1}(c)} nov(\gamma)$$

where

$$ nov(\gamma) = \left\{\begin{array}{l} 0 \text{, if } \exists  C_{i} \in V, \gamma \in C_{i} \\ 1 \text{, otherwise} \end{array}\right. $$

Intuitively, a coordinate is novel if it has never appeared in *V* and not novel otherwise. The novelty of a component is the average novelty of its coordinates.

### Peculiarity

Estimating peculiarity requires first of all to define the concept of “corresponding cell(s)” of each coordinate *γ* of *C* in the cube $C^{\prime }$ obtained by the last intention the user has formulated with *C*_0_, which is done through a *proxy* function as follows. Intuitively, if the intention changes the group-by set, the corresponding coordinates(s) of *γ* are determined via the part-of order; if the intention changes the selection predicate, the corresponding coordinates of *γ* are *γ* itself if it is part of $C^{\prime }$, the empty set otherwise; if the intention changes the measure, the corresponding coordinates of *γ* are the empty set.

#### **Definition 9** (Proxies)

Let *C* be a cube over cube schema $\mathcal {C}$, and $C^{\prime }$ be the cube occurring immediately before *C* in the current session *V*. Let *γ* be a coordinate of *C*, and *m* be a measure in *C*. The *proxies* of *γ* for *m* are defined as

$$ proxy_{C,m}(\gamma) = \left\{\begin{array}{l} \{\gamma^{\prime} : \gamma^{\prime} \in C^{\prime}, \gamma^{\prime} \lesseqgtr \gamma \}, \text{if}~ m ~\text{is in}~ C^{\prime}\\ \varnothing \text{, otherwise} \end{array}\right. $$

For the first intention in an analysis session, $C^{\prime }$ is undefined; since in this case the user has no prior belief, we conventionally put $proxy_{C,m}(\gamma ) = \varnothing $ for all *γ* ∈ *C*.

Note that, in OLAP terms, if *C* is a roll-up of $C^{\prime }$, the inter-cells mapping defined by the proxy function is many-to-one; if *C* is a drill-down of $C^{\prime }$, the mapping is one-to-many; in all other cases (*drill-anywhere*), the mapping is many-to-many.

#### *Example 6*

**Fig. 5:**
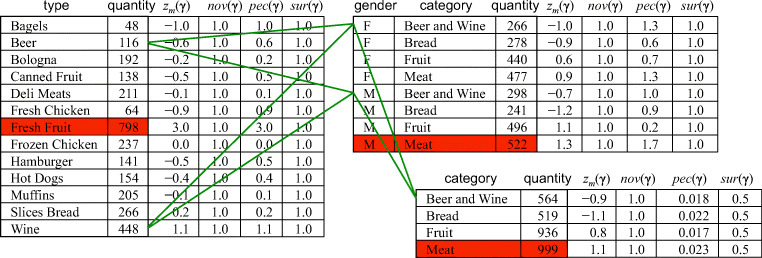
Cubes *C*_1_ (left), *C*_2_ (top-right), and *C*_3_ (bottom-right) in Example 6; in red the highlights for the top-1 model, in green some of the proxy inter-cell relationships

Let

$$ \begin{array}{@{}rcl@{}} &&\textsf{with SALES describe quantity}\\ &&  \textsf{for month = '1997-04' by type}\\ &&\textsf{with SALES describe quantity}\\ &&  \textsf{for month = '1997-04' by gender,category}\\ &&  \textsf{using top-k size 1}\\ &&\textsf{with SALES describe quantity}\\ &&  \textsf{for month = '1997-04' by category}\\ &&  \textsf{using top-k size 1} \end{array} $$

be a sequence of three intentions *q*_1_, *q*_2_, *q*_3_ formulated by the user. When no level is specified in the by clause for hierarchy *h*, it is implicitly assumed by*ALL*_*h*_. Thus, while the plan generated for the first intention relies on query *q*_1_ = *q* defined in Example 4, the ones for the second and third intentions rely on *q*_2_ and *q*_3_ with $G_{q_{1}} = \{\textsf {allDates},\textsf {gender},\textsf {category},\textsf {allStores}\}$ and $G_{q_{2}} = \{\textsf {allDates},\textsf {allCustomers},\textsf {category},\textsf {allStores}\}$, respectively (the selection predicates and measures do not change). Let *C*_1_, *C*_2_, and *C*_3_ be the cubes resulting from *q*_1_, *q*_2_, and *q*_3_, respectively. Some of the inter-cell relationships induced by the proxy function are shown by green lines in Fig. 5. Since *C*_2_ is a drill-anywhere of *C*_1_, the relationship is many-to-many; conversely, since *C*_3_ is a roll-up of *C*_2_, the relationship here is many-to-one.

We can now define peculiarity as follows.

#### **Definition 10** (Peculiarity)

Let *c* be a component of model ${\mathscr{M}}$. The *peculiarity* of *c* is defined as

$$pec(c)=\frac{avg_{\gamma \in \mu^{-1}(c)} pec(\gamma)}{max_{\gamma \in C}pec(\gamma)} $$

where

$$ pec(\gamma) = max_{m \in C} (|z_{m}(\gamma) - avg_{\gamma^{\prime} \in proxy_{C,m}(\gamma)} z_{m}(\gamma^{\prime})|) $$

and function *z*_*m*_() returns the z-score of a cell for measure *m* over the whole cube that the cell belongs to.

Intuitively, the z-score captures to what extent the value of a measure for a cell deviates from the measure values for other cells in the cube, and peculiarity compares the z-scores of a cell with those of its proxies. A cell is more peculiar if such difference is higher. The peculiarity of a component is the average peculiarity of its coordinates, normalized by the highest peculiarity value.

#### *Example 7*

Consider again the intentions in Example 6. Figure 5 shows the z-score, the novelty, and the peculiarity of each cell of the three cubes. The novelty is 1 for all cells, since in all cases the coordinates are seen for the first time during the session. As to the peculiarity, in *C*_1_ its values are simply the absolute values of the z-scores *z*_*m*_, as per Definition 10 (*C*_1_ is the result of the first intention in the session, so the set of proxies is empty for all coordinates).

### Surprise

While novelty describes whether a cell was previously unknown to the user (i.e., not present in *V* ), surprise assesses whether it challenges the user’s previous beliefs (i.e., what the user learned from *V* ).

#### **Definition 11** (Surprise)

Let *c* be a component of model ${\mathscr{M}}$. The *surprise* of *c* is defined as

$$ sur(c)=avg_{\gamma \in \mu^{-1}(c)} sur(\gamma) $$

where

$$ sur(\gamma) = 1- \frac{avg_{u \in \gamma} (| \{C_{i} \in V : u \in \gamma_{i}, \gamma_{i} \in C_{i} \}|)}{|V|} $$

Intuitively, a coordinate is more surprising if its members were not frequently seen in *V*. Hence, we count the number of cubes each member appears in; the surprise of coordinate *γ* is 0 when all of its members already appeared in all the cubes of *V*, 1 when all of its members never appeared in *V*. For the first intention in an analysis session, we set *sur*(*c*) = 1 for all components *c*. The surprise of a component is the average surprise of its coordinates.

Note that novelty and surprise are defined in a such way that a coordinate can be novel and still have a low surprise (if all its members are frequent in *V* ) and, conversely, a coordinate can be surprising while not being novel (for instance if it was seen only once and all its members are infrequent in *V* ).

#### *Example 8*

Consider again the intentions in Example 6. Figure 5 shows the surprise of each cell of the three cubes. Note that for *C*_1_ and *C*_2_ all cells have surprise 1, since all the members of their coordinates were never seen before. Conversely, the cells of *C*_3_ have surprise 0.5, since each of their members was already seen once within a history of two previous cubes (|*V* | = 2). Now, let ${\mathscr{M}}_{2}$ be the model of type top-k, with *k* = 1, computed on *C*_2_; this model has two components: ${c_{2}^{1}}$, including only the top-1 cell (in red), and ${c_{2}^{2}}$, including all the others. The interestingness values for these two components are $interest({c_{2}^{1}})=1.00$ and $interest({c_{2}^{2}})=0.83$, respectively. So, the enhanced cube *E*_2_ resulting from the second intention includes *C*_2_, ${\mathscr{M}}_{2}$, and the highlight ${c_{2}^{1}}$. Finally, let ${\mathscr{M}}_{3}$ be the top-1 model computed on *C*_2_, with components ${c_{3}^{1}}$ (the top-1 cell, in red) and ${c_{3}^{2}}$ (all the other cells). It is $interest({c_{3}^{1}})=0.83$ and $interest({c_{3}^{2}})=78$, so the highlight here is ${c_{3}^{1}}$.

#### *Example 9*

As an example of computation of interestingness when an intention changes the selection predicate of the previous one, consider the session

$$ \begin{array}{@{}rcl@{}} &&\textsf{with SALES describe quantity}\\ &&  \textsf{for type = 'Beer' by product}\\ &&\textsf{with SALES describe quantity}\\ &&  \textsf{for category = 'Beer and Wine' by product} \end{array} $$

**Fig. 6:**
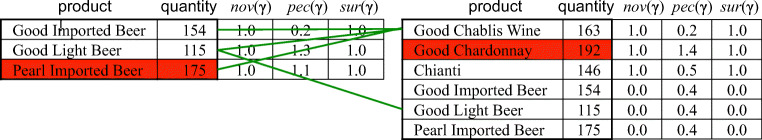
Cubes *C*_1_ (left) and *C*_2_ (right) in Example 9; in red the highlight for the top-1 model, in green some of the proxy inter-cell relationships

The resulting cubes are shown in Fig. 6. Here, the proxy mapping for the cells included in both cubes is one-to-one; conversely, the cells in *C*_2_ that were not present in *C*_1_ map to all the cells of *C*_1_.

## Execution Plans for describe Intentions

The describe operator provides an answer to the user asking “show me my business” by describing one or more cube measures, possibly focused on one or more level members, at some given granularity (Vassiliadis et al. 2019). The cube is enhanced by showing either the top/bottom-k cells, the skyline, the outliers, or clusters of cells.

Let *C*_0_ be a base cube over cube schema $\mathcal {C}=(H, M)$. The general syntax for describe is

$$ \begin{array}{@{}rcl@{}} &&\textsf{with $C_{0}$ describe $m_{1}, \ldots, m_{z}$}\\ &&  \textsf{[ for \textit{P} ] [ by $l_{1}, \ldots,l_{n}$ ]}\\ &&  \textsf{[ using $t_{1}$ [ size $k_{1}$ ], $\ldots, t_{r}$ [ size $k_{r}$ ]]} \end{array} $$

(optional parts are in brackets) where *m*_1_,…, *m*_*z*_ ∈ *M* are measures of $\mathcal {C}$, *P* is a set of selection predicates each over one level of *H*, {*l*_1_,…, *l*_*n*_} denote a group-by set of *H*,^4^*t*_1_,…, *t*_*r*_ are model types, and the *k*_*i*_’s are the desired sizes to be applied to the models returned as explained in point 2 below.

The plan corresponding to a fully-specified intention, i.e., one where all optional clauses have been specified, is:

1. Execute query *q* = (*G*_*q*_, *P*_*q*_, *M*_*q*_), where *G*_*q*_ = {*l*_1_,…, *l*_*n*_}, *P*_*q*_ = *P*, and *M*_*q*_ = {*m*_1_,…, *m*_*z*_}. Let *C* = *q*(*C*_0_) be the cube resulting from the execution of *q* over *C*_0_.
2. For 1 ≤ *i* ≤ *r*, compute model ${\mathscr{M}}_{i}=(t_{i},alg_{i},C,In_{i},Out_{i},\mu _{i})$ and for each *c* ∈ *Out*_*i*_, compute *interest*(*c*). Size *k*_*i*_ is used for clustering to determine the number of clusters to be computed, for top-k and bottom-k to determine the number of cells to be returned, for outliers to determine the number of outliers; it is neglected for the skyline.
3. Find the highlight $c_{high} = argmax_{\{c \in \bigcup _{i} Out_{i}\}}$ (*interest*(*c*)).
4. Return the enhanced cube *E* consisting of *C*, $\{{\mathscr{M}}_{1}, {\ldots } {\mathscr{M}}_{r} \}$, and highlight *c*_*high*_.

Partially-specified intentions are interpreted as follows:

- If the for clause has not been specified, we consider *P*_*q*_ = *TRUE*.
- If the by clause has not been specified, we consider *G*_*q*_ = {*ALL*_1_,…, *ALL*_*n*_}.
- If the using *t*_1_,…, *t*_*r*_ clause has not been specified, all model types listed in Section 3 are computed over *C* (the skyline is computed only if *z* > 1, i.e., at least two measures have been specified).
- If the size clause has not been specified for one or more models, the value of *k*_*i*_ is determined automatically as discussed in Section 6.

### *Example 10*

Consider the following session on the SALES cube:

$$ \begin{array}{@{}rcl@{}} &&\textsf{with SALES describe quantity}\\ &&  \textsf{for month = '1997-04' by type} \\ &&\textsf{with SALES describe quantity}\\ &&  \textsf{by category}\\ &&  \textsf{using clustering size 3} \\ &&\textsf{with SALES describe quantity, storeSales}\\ &&  \textsf{for country = 'Italy'}\\ &&  \textsf{using skyline} \end{array} $$

The models computed for the first intention are top-k, bottom-k, clustering, and outliers (computing the skyline for a single measure makes no sense). For the second and the third intentions, a clustering producing 3 clusters and the skyline are computed, respectively.

## Setting the Model Size

Our approach to find the best value for the size parameter *k* when it is not specified in the intention is based on good practices in hierarchical clustering, especially when single-linkage is used, meaning that inter-cluster distance is measured by the closest two points of the clusters. The best separation of clusters can then be found by finding the knee of the evaluation graph of the clustering algorithm, which is a two dimensional plot where the x-axis is the number of clusters produced and the y-axis is one classical clustering evaluation metric (error, silhouette, etc.) considering *x* clusters. In hierarchical clustering, since the cost for merging clusters constantly increases, the evaluation graph often looks like an L-shaped curve with a more or less defined knee. The assumption usually made is that the best merging cost threshold is at the curve knee, where the curve switches from a sharp slope to a low decreasing line.

We tested two solutions from the literature, namely L-method (Salvador and Chan 2004) and Kneedle (Satopaa et al. 2011), which have been proposed to find the knee in a curve of discrete data. These methods were compared using 3-dimensional non-random toy datasets specifically created for the experiment with the *Scikit-Learn* Python package, varying the size (6, 30, and 300 samples) and the shape of clusters, defining a ground truth. We only report the main findings.

**Fig. 7:**
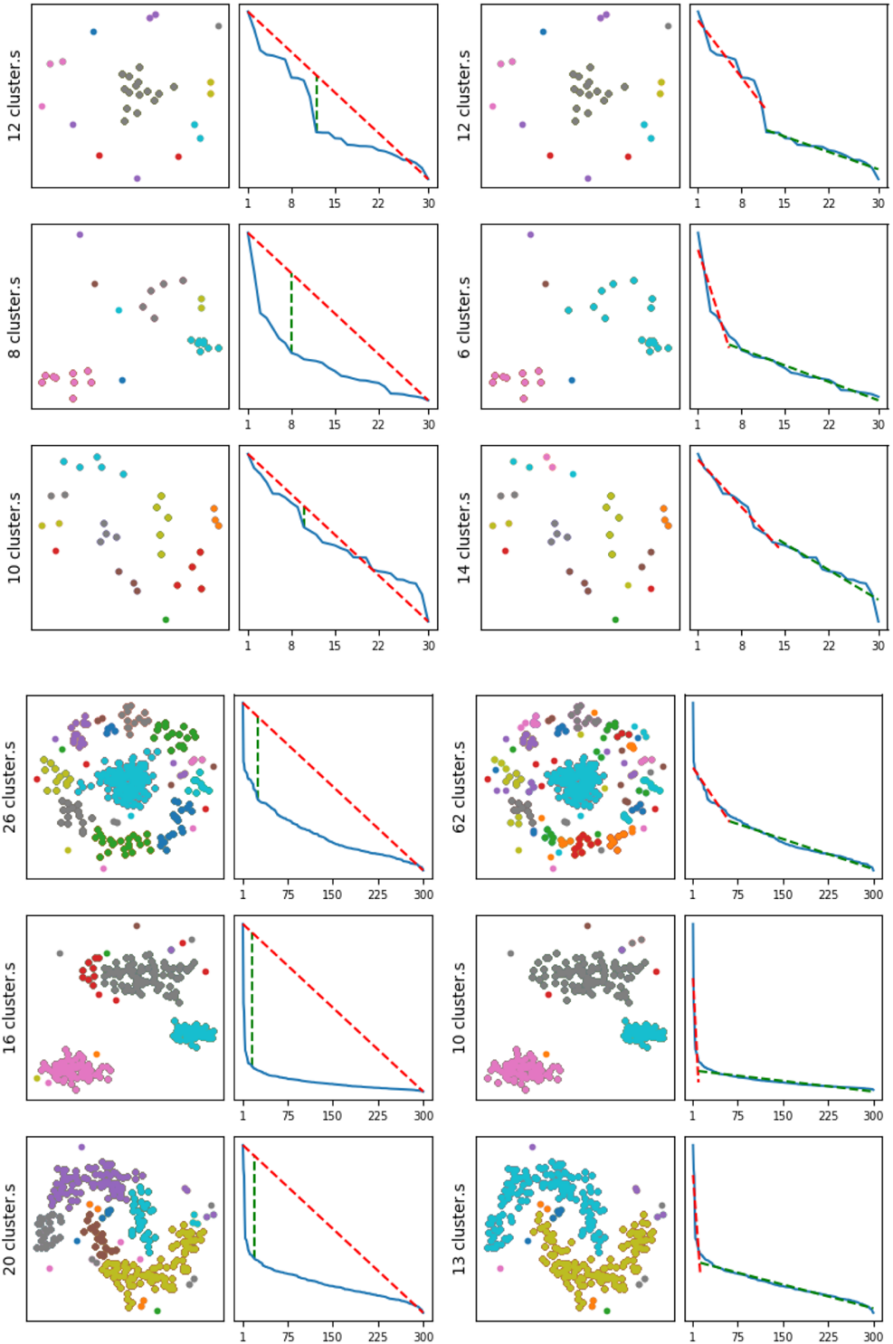
Results on the 30 (left) and 300 (right) samples data for Kneedle (columns 1 and 3) and L-method (columns 2 and 4)

While both methods achieve similar good results for knee detection, the L-method takes longer to execute and tends to shift the knee on large data sets. This can be seen, for instance, in Fig. 7 on the top-right graph. The right knee seems to be located at *x* = 25 but the method returned a knee at *x* = 62. Since Kneedle is quicker and provides more consistent results, we have adopted it to determine *k*, both for clustering (*k* being the number of clusters), top/bottom-k (where *k* is the number of points in the first cluster, i.e., the one with higher values), and outliers (where *k* is the number of points in the first and last cluster).

## Visualizing Enhanced Cubes

In this section we discuss how to provide an effective description of an enhanced cube by coupling text-based representations (a pivot table and a ranked component list) and graphical representations (one or more charts) with an ad-hoc interaction paradigm. The guidelines we adopt to this end are explained below:

(i) For visualization purposes, we assume that an intention can select at most three measures (1 ≤ *z* ≤ 3) and three group-by levels (1 ≤ *n* ≤ 3). This is actually not a strong limitation, considering that a visualization of four or more dimensions and/or measures using a single table or chart is hardly interpretable and definitely not intuitive.
(ii) Since we are focusing on intentions aimed at *describing* data, we believe that providing multiple visualizations from different points of view should be preferred to just picking the “most effective one”. Indeed, the effectiveness of a visualization type largely depends on the skills and personal tastes of each user.
(iii) We restrict to considering visualization types that can be easily understood both by lay users and skilled users, and are suitable for multidimensional data.
(iv) Clearly, the effectiveness of a visualization type also depends on the features of the specific dataset. Using an unsuitable visualization can generate confusion and misunderstandings in users, and can lead them to wrong conclusions. Thus, for each intention we visualize only the charts that are recognized to be suitable given the characteristics of the data to be shown.
(v) Models and components play a key role in the IAM approach. Thus, the visualizations we provide aims at showing not only dimension and measure values, but also the different components of a model using a color code. For the same reason, the interaction paradigm should be component-driven.

The visualization we provide for enhanced cube *E* based on guidelines (ii) and (v) includes three distinct but inter-related areas: a *table* area that shows the cube cells using a pivot table; a *chart* area that complements the table area by representing the cube cells through one or more charts; a *component* area that shows a list of model components sorted by their interestingness. The chart types we consider following guidelines (i) and (iii) are multiple line graphs, radar charts, grouped column charts, heat maps, bubble charts, parallel coordinate charts, and scatter plots. The heuristics we adopt to decide whether using or not each chart type for a given enhanced cube *E* (guideline (iv)) was inspired by the work of Golfarelli and Rizzi (2020), where a *suitability score* is assigned to each chart type depending on the features of the dataset to be visualized. For instance, bubble charts are considered to be suitable to visualize *n*-dimensional data if the bubble size is mapped to a numerical attribute — such as a measure — and the bubble color is mapped to either a numerical attribute — such as a second measure — or a categorical attribute — such as a model component. Specifically, the features of *E* we take into account to this end are the number *n* of dimensions, the number *z* of measures, and the domain cardinality and type of the dimensions.

**Fig. 8:**
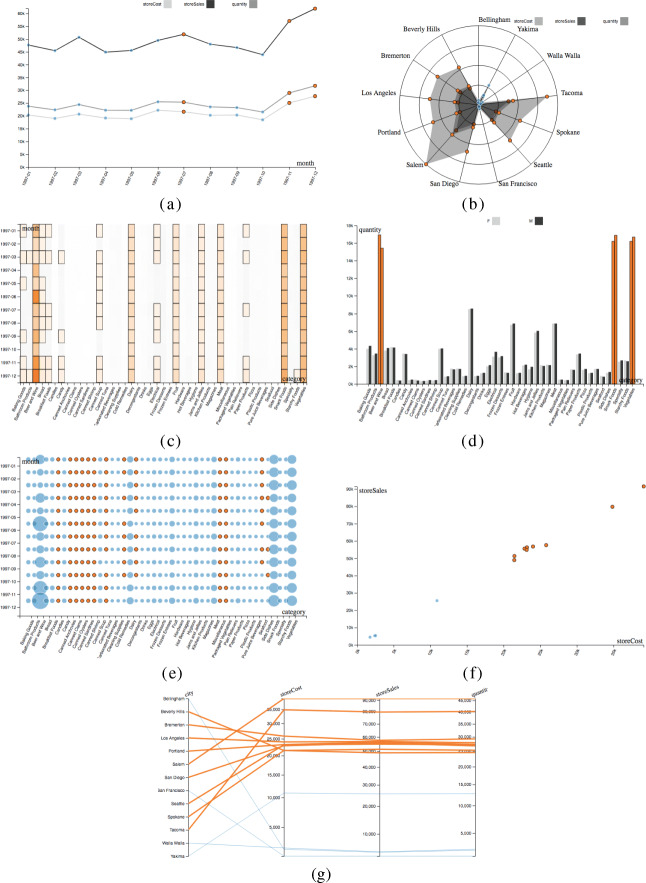
Chart types: multiple line graph (a), radar chart (b), heat map (c), grouped column chart (d), bubble chart (e), scatter plot (f), and parallel line chart (g); in orange and blue, the different components of the related models

The pseudocode is shown in Algorithm 1; it is based on the heuristics described below:

- If *E* has one dimension *d*_1_ (of temporal type) and one or more measures, draw a **multiple line graph** using the X axis for *d*_1_ and the Y axis for the measure(s) values (Fig. 8a). Different line colors are used to distinguish the different measures. Markers take the colors of the components of model *t*, i.e., the model to which the highlight of *E* belongs.
- If *E* has one low-cardinality dimension *d*_1_ (of non-temporal type) and one or more measures, draw a **radar chart** using the angle for *d*_1_ and the radius for measure(s) values (Fig. 8b). Different line colors are used to distinguish the different measures. Markers take the colors of the components of *t*.
- If *E* has one dimension *d*_1_ and one or more measures, draw a **heat map** using the X axis for *d*_1_ and the Y axis for the different measures (Fig. 8c). Measure(s) values are shown using shades of color.
- If *E* has two low-cardinality dimensions *d*_1_, *d*_2_ and one measure, draw a **grouped column chart** using the X axis for *d*_1_, the Y axis for measure values, and the color for *d*_2_ (Fig. 8d).
- If *E* has two dimensions *d*_1_, *d*_2_ and one measure, draw a **heat map** using the X axis for *d*_1_, the Y axis for *d*_2_, and the color shades for measure values.
- If *E* has two (three) dimensions *d*_1_, *d*_2_ (*d*_3_) and one or two measures, draw a 2D (3D) **bubble chart** using the X axis for *d*_1_, the Y axis for *d*_2_, (the Z axis for *d*_3_), and the bubble size for the values of one measure (Fig. 8e). If there is a second measure, its values are shown using shades of color of bubbles; otherwise, bubbles take the colors of the components of *t*.
- If *E* has two (three) measures, draw a 2D (3D) **scatter plot** using the X, Y (Z) axes for the different measures (Fig. 8f). Points take the colors of the components of *t*.
- If *E* has three measures, draw a **parallel coordinate chart** using one coordinate for each measure (Fig. 8g). Lines take the colors of the components of *t*.

A summary of the chart types used depending on the number of dimensions *n* and the number of measures *z* is shown in Table 1.

**Figure:**
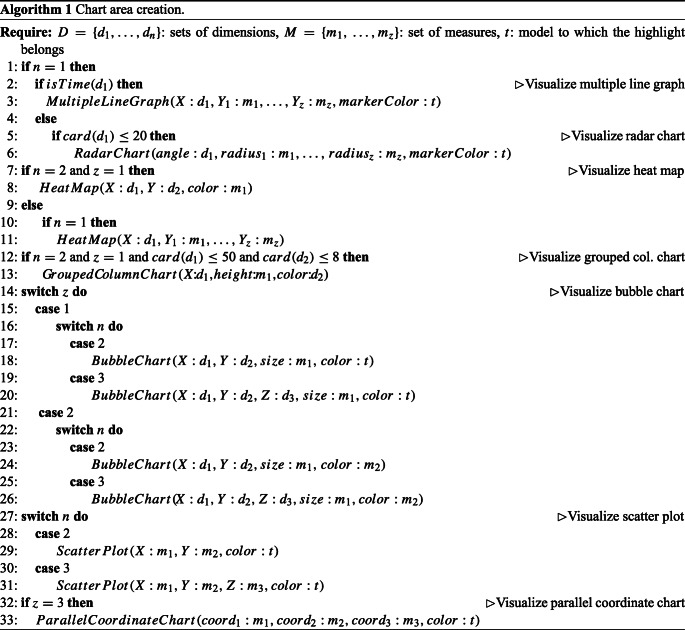

**Table 1:** Summary of chart types used depending on the number of dimensions *n* and the number of measures *z* (MLC = multiple line chart, RC = radar chart, HM = heat map, SP = scatter plot, PCC = parallel coordinate chart, GCC = grouped column chart, BC = bubble chart)

	*z* = 1	*z* = 2	*z* = 3
*n* = 1	MLC/RC, HM	MLC/RC, HM, SP	MLC/RC, HM,
			SP, PCC
*n* = 2	GCC (low card.),	BC, SP	SP, PCC
	HM, BC		
*n* = 3	BC	BC, SP	SP, PCC

The interaction paradigm we adopt is component-driven (guideline (v)). Specifically, clicking on one component *c* in the component area leads to emphasizing the corresponding cube cells (i.e., those that map to *c* via function *μ*) both in the table area and in the chart area. The highlight is the top component in the list and is selected by default. Following the *details-on-demand* paradigm (Shneiderman 1996), interaction is enhanced using a tooltip that, when the mouse is positioned on a data point, shows its coordinate, its measure value(s), and the component(s) it belongs to.

### *Example 11*

**Fig. 9:**
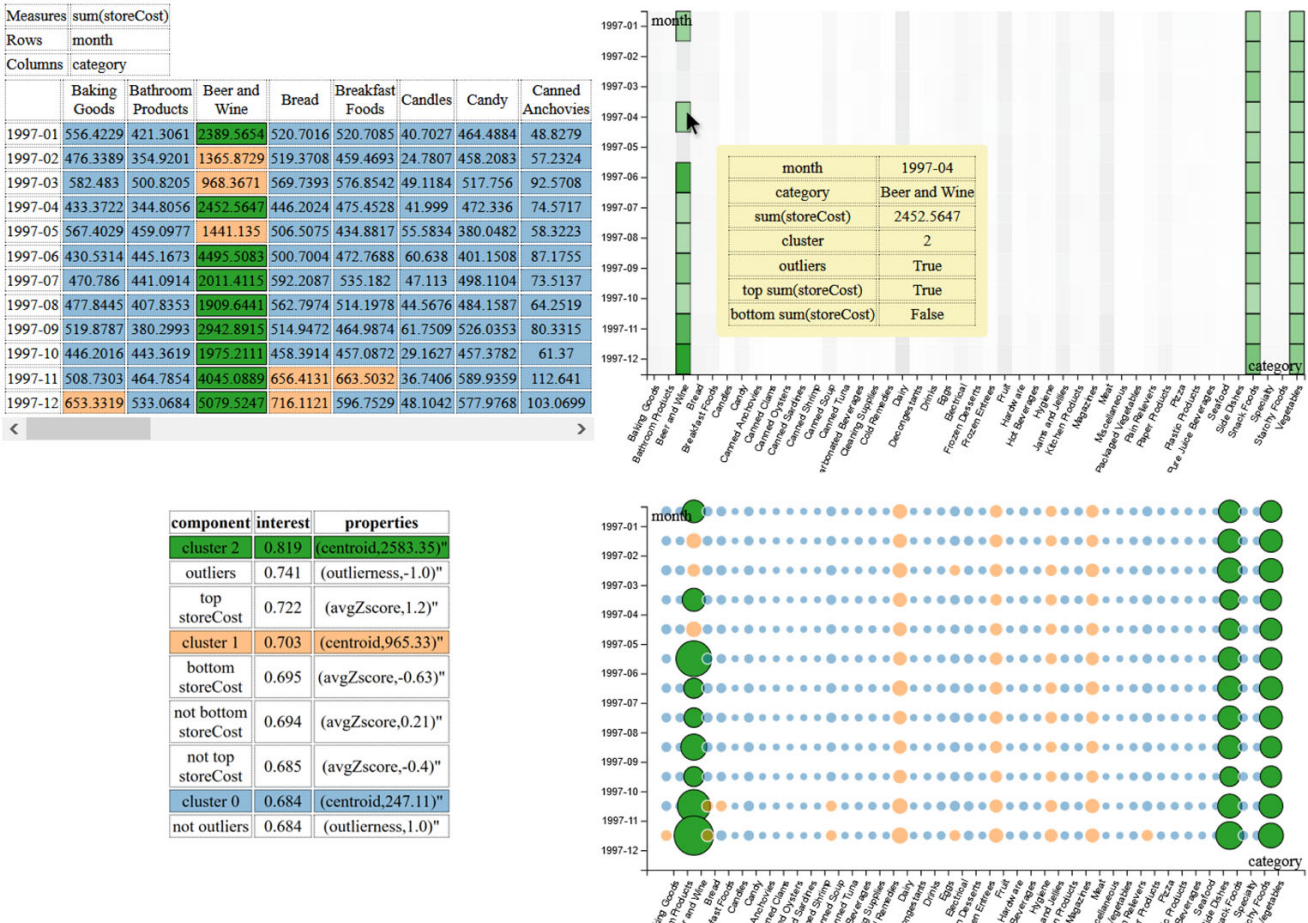
The visualization obtained for the intention in Example 11

Figure 9 shows the visualization obtained when the following intention is formulated:

$$ \begin{array}{@{}rcl@{}} &&\textsf{with SALES describe storeCost}\\ &&  \textsf{by month, category} \end{array} $$

On the top-left, the table area; on the right, the chart area; on the bottom-left, the component area. Here it is *n* = 2 and *z* = 1, so a heat map and a bubble chart have been selected (the grouped column chart is not selected due to the high cardinality of the month dimension). The top-interestingness component is a cluster, so a color has been assigned to each component of clustering (i.e., to each cluster) and is uniformly used in all three areas. The highlight (in green) is currently selected and is emphasized using a thicker border in all areas. Note that a tooltip with all the details about a single cell is also shown (in yellow).

## Experimental Tests

In this section we discuss the results of the tests we made to evaluate our approach from four points of view: formulation effort (as compared to the one using plain SQL and Python), effectiveness (as compared to the interestingness measure used by Chédin et al. (2020)), efficiency, and scalability. The prototype implementation we used for the tests uses the simple multidimensional engine described by Francia et al. (2020), which in turn relies on the Oracle 11g DBMS to execute queries on a star schema based on multidimensional metadata (in principle, the prototype could work on top of any other multidimensional engine). The mining models are imported from the Scikit-Learn Python library. Finally, the web-based visualization is implemented in JavaScript and exploits the D3 library for chart visualization. The prototype implementation can be accessed at http://semantic.csr.unibo.it/describe/.

### Formulation Effort

The first goal of our experiments is to evaluate the saving in user’s effort when writing a describe intention over the one necessary to obtain the same result using plain SQL and Python. To this end we adopt the simple metric proposed by Jain et al. (2016), where the ASCII character length is used as an approximation for the effort it takes to craft a query.^5^

For this evaluation we used a simple session including three intentions on the SALES cube, where the by clause is progressively enlarged and all the models are computed:

$$ \begin{array}{@{}rcl@{}} I_{1} &: &\textsf{with SALES describe quantity, revenue}\\ &&  \textsf{by date}\\ I_{2} &: &\textsf{with SALES describe quantity, revenue}\\ &&  \textsf{by date, customer}\\ I_{3} &: &\textsf{with SALES describe quantity, revenue}\\ &&  \textsf{by date, customer, product} \end{array} $$

The results are shown in Table 2; for SQL and Python we considered the code generated by our prototype to execute each intention. Remarkably, the total formulation effort using SQL+Python is, for each intention type, almost two orders of magnitude larger than using describe intentions.

**Table 2:** Formulation effort for different intentions (numbers of characters)

Intention	SQL	Python	Total	describe
*I*_1_	234	5038	5272	45
*I*_2_	361	5038	5399	55
*I*_3_	478	5038	5516	64

To also have some insight into the time required to operate manually, we asked five PhD students in computer science to use Python to manually extract two types of models (outliers and clustering) from a 2000 tuples bidimensional cube. This real-world cube was created from the COVID dataset made available by the European Center for Disease Prevention and Control.^6^ Table 3 shows, for each student, her skill in Python (beginner/intermediate/advanced), the time taken for doing the exercise (in minutes), the models she extracted, and the ASCII character length of the Python code she wrote, disregarding the quality of the models extracted. We remark that even skilled students needed quite a long time for extracting both models, and had to write substantial Python programs (even though, in comparison with Table 2, they were asked to compute two models only).

**Table 3:** Time (minutes) and formulation effort (numbers of characters for manual model extraction

Student Id	Skill	Time	Models	Length
A	advanced	45	clustering	3479
B	advanced	51	both	1777
C	intermediate	25	outliers	935
D	advanced	59	both	1150
E	advanced	90	outliers	2627

### Effectiveness

Our second experimental goal is to assess the effectiveness of our approach. Specifically, we compare the 3-facets interestingness measure as of Definition 7 with the 1-facet measure adopted by Chédin et al. (2020); note that the latter mostly corresponds to peculiarity as of Definition 10. The experimental setting we use here is again that of a real-world cube extracted from the COVID dataset. On this cube we run 20 distinct describe sessions (including exactly 7 intentions each), of which 10 were created manually as done by Outa et al. (2020), and 10 were created with the CubeLoad workload generator (Rizzi and Gallinucci 2014).

To compare the two interestingness measures we compute the *highlight coverage* of each intention *I* as follows. Let *C*_0_ be the base cube and *c* be the highlight of *I*; we define the coverage of *c* as

$$ cov(c) = \frac{|\{\gamma \in C_{0} : \exists \gamma^{\prime} \in \mu^{-1}(c), \gamma^{\prime} \lesseqgtr \gamma\}|}{|C_{0}|} $$

**Fig. 10:**
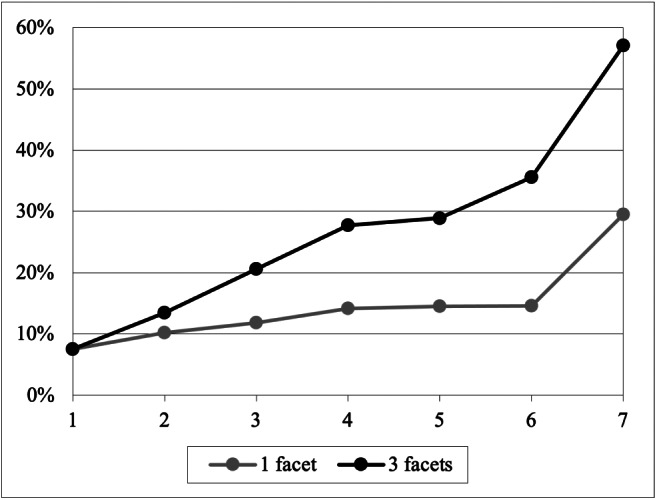
Average cumulative highlight coverage at different session steps for the 1-facet and 3-facets interestingness measures

Intuitively, the coverage of highlight *c* is the percentage of cells of *C*_0_ that roll-up to cells belonging to *c*. The cumulative highlight coverages at each session step, averaged over all 20 sessions, are reported in Fig. 10 (all *α* weights in Definition 7 are set to $\frac {1}{3}$).

Overall, the figure clearly shows that the cumulative coverage of the 3-facets interestingness is higher than the one of the 1-facet interestingness, which means that the enhanced formulation we adopted in this work is more effective in providing diversified highlights over the cube, leading to a more comprehensive exploration. We also noted that the by clause has a major impact on the highlights, i.e., in sessions mainly consisting of roll-ups and drill-downs the two measures of interestingness behave quite similarly since peculiarity is the main driver. On the other hand, the longer the session, the larger the effect of surprise and novelty in ensuring a more diversified coverage.

### Efficiency

Our third experimental goal is to investigate if the performance of our approach is compatible with the near-real-time requirement of interactive analysis sessions. To this end we populated the SALES cube using the FoodMart data.^7^ We reused the 3-intention session introduced in Section 8.1; from the performance point of view this corresponds to considering the worst case, in which all five models are computed on cubes obtained by progressively including in the group-by set the three dimensions with highest cardinality. The tests were run on an Intel(R) Core(TM)i7-6700 CPU@3.40GHz with 8GB RAM.

Table 4 shows the total execution time and its breakdown into the times necessary to query the base cube, to compute the models, to measure the interestingness, and to generate the pivot table returned to the browser. Remarkably, it turns out that at most 18 seconds are necessary to retrieve and visualize an enhanced cube of more than 86000 cells, which is perfectly compatible with the execution time of a normal OLAP query. The table shows that the main cost component is, after model computation, the measurement of interestingness. The most computationally-expensive facets are peculiarity and surprise, the former mostly depending on the cube cardinality, the latter increasing with the session length.

**Table 4:** Execution times in seconds for three intentions with increasing cardinalities of *C*

Intention	∣*C*∣	Query	Model	Interestingness	Pivot	Total
*I*_1_	323	0.10	0.25	0.00	0.00	0.36
*I*_2_	20525	0.22	5.90	0.36	0.36	6.83
*I*_3_	86832	0.22	8.50	7.43	1.72	17.87

### Scalability

Our last experimental goal is to evaluate the scalability of our approach. To this end we used the Star Schema Benchmark (SSB) cube, described by four hierarchies; please refer to the work by O’Neil et al. (2009) for the logical schema of the SSB dataset. Specifically, we generated three base SSB cubes, namely *SSB*_1_, *SSB*_10_, and *SSB*_100_, with different scale factors resulting in the following cardinalities:

$$ \begin{array}{@{}rcl@{}} |SSB_{1}|&=&6 \cdot 10^{6}\\ |SSB_{10}|&=&6 \cdot 10^{7}\\ |SSB_{100}|&=&6 \cdot 10^{8} \end{array} $$

Note that the cardinality of each cube is equal to the number of tuples in the corresponding fact table. As commonly done in OLAP settings, primary and foreign keys were indexed using B-Trees, and materialized views were created to improve performances.

The experiments were focused on three describe intentions similar to those introduced in Section 8.1, i.e., with progressively-enlarged group-by sets. Since the by and for clauses of each describe intention are not changed, scaling up the cardinality of the base cube implies that also the cardinality of the resulting cube *C* scales up as shown in Table 5. To reduce the impact of caching, each intention was executed five times on each base cube, and the execution times were averaged.

**Table 5:** Resulting cardinalities of *C* for each intention applied to each base cube

Intention	*SSB*_1_	*SSB*_10_	*SSB*_100_
*I*_1_	7	7	7
*I*_2_	35	35	35
*I*_3_	13300	139020	1396955

**Fig. 11:**
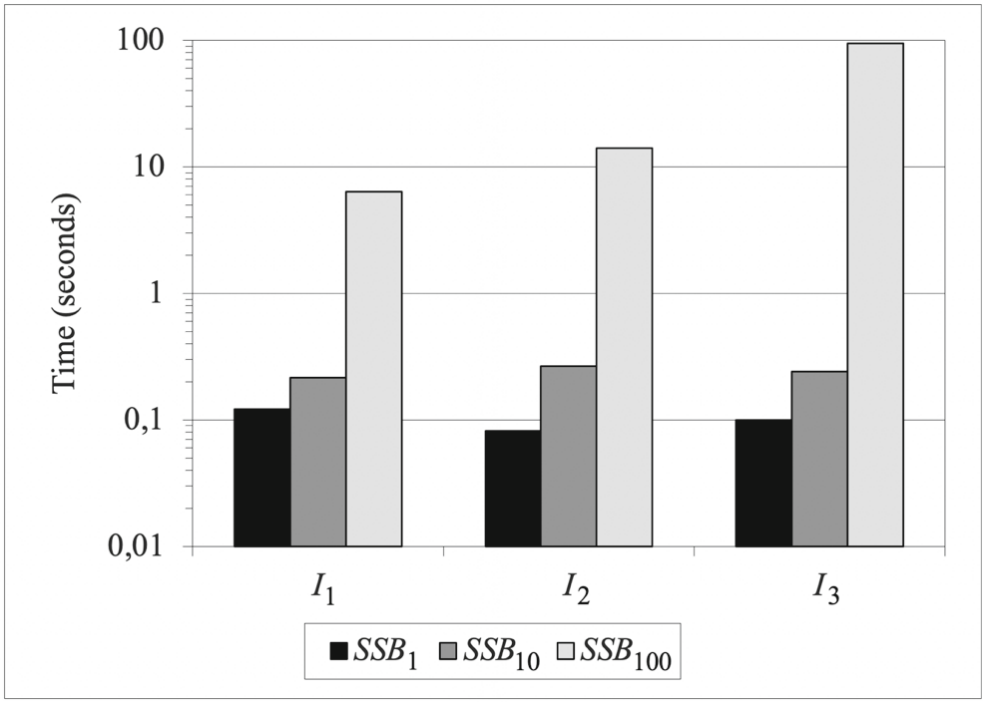
Execution times for increasing cardinalities of the base cube

Figure 11 shows, on a logarithmic scale, the times in seconds for executing the three intentions on the three base cubes with increasing cardinalities. When *I*_3_ is executed over *SSB*_100_, yielding as a result a cube with almost 1.5 millions of cells, the overall time turns out to be about 95 seconds, which is still compatible with the requirements of an interactive analysis session. Of this time, 68 seconds are used to compute the models, and 24 seconds to compute the interestingness. Though the chart shows an exponential trend, which clearly raises some concerns about further scalability, we observe that even dealing with a 1.5M-cells cube should be considered quite unusual in the context of an analysis session.

## Related Work

The idea of coupling data and analytical models was born in the 90’s with inductive databases, where data were coupled with patterns meant as generalizations of the data (Raedt 2002). Later on, data-to-model unification was addressed in MauveDB (Deshpande and Madden 2006), which provides a language for specifying model-based views of data using common statistical models. However, achieving a unified view of data and models was still seen as a research challenge in business intelligence a few years later (Pedersen 2009). More recently, Northstar (Kraska 2018) has been proposed as a system to support interactive data science by enabling users to switch between data exploration and model building, adopting a real-time strategy for hyper-parameter tuning. Finally, the coupling of data and models is at the core of the IAM vision (Vassiliadis et al. 2019), on which this paper relies. The three basic pillars of IAM are (i) the redefinition of query as expressing the user’s intention rather than explicitly declaring what data are to be retrieved, (ii) the extension of query results from plain data cubes to cubes enhanced with models and highlights, and (iii) the characterization of model components in terms of their interestingness to users.

The coupling of the OLAP paradigm and data mining to create an approach where concise patterns are extracted from multidimensional data for user’s evaluation, was the goal of some approaches commonly labeled as OLAM (Han 1997). In this context, k-means clustering is used by Bentayeb and Favre (2009) to dynamically create semantically-rich aggregates of facts other than those statically provided by dimension hierarchies. Similarly, the shrink operator is proposed by Golfarelli et al. (2014) to compute small-size approximations of a cube via agglomerative clustering. Other operators that enrich data with knowledge extraction results are DIFF (Sarawagi 1999), which returns a set of tuples that most successfully describe the difference of values between two cells of a cube, and RELAX (Sathe and Sarawagi 2001), which verifies whether a pattern observed at a certain level of detail is also present at a coarser level of detail, too. Finally, Chen et al. (2005) reuse the OLAP paradigm to explore prediction cubes, i.e., cubes where each cell summarizes a predictive model trained on the data corresponding to that cell. The IAM approach can be regarded as OLAM since, like the approaches mentioned above, it relies on mining techniques to enhance the cube resulting from an OLAP query. However, while each of the approaches above uses one single technique (e.g., clustering) to this end, the IAM leans on multiple mining techniques to give users a wider variety of insights, using the interestingness measure to select the most relevant ones.

In the same direction, Sarawagi (2000) describes a method that profiles the exploration of a user and uses the Maximum Entropy principle to recommend which unvisited parts of the cube can be the most surprising in a subsequent query. The Cinecubes method (Gkesoulis and Vassiliadis 2013; Gkesoulis et al. 2015) aims at providing automated reporting as a result to an original OLAP query. The proposed method enriches an original OLAP query with auxiliary queries to aid (a) the comparison and assessment of the result of the query to similar data and (b) the explanation of the result with values at the most detailed level. So, the results of the Cinecubes system can coarsely be grouped as the result of two operators: the first one computes queries for values similar to ones defining the selection filters of the original query; the second one by drilling down into the dimensions of the result, one dimension at a time.

The characteristics of the different approaches for visualizing data and interacting with them have been deeply explored in the literature, also with reference to their suitability for datasets with different features and users with varying skills and goals. Börner (2015) surveys the classifications proposed in the literature for visualization types and integrates them into a single comprehensive framework. Abela (2008) proposes a decision tree to select the best visualization according to the user’s goal and to the main features of data. More recently, SkyViz — to which our approach is inspired — starts from a visualization context based on seven coordinates for assessing the user’s objectives and describing the data to be visualized (Golfarelli and Rizzi 2020). Then it uses skyline-based techniques to translate a visualization context into a set of suitable visualization types and to find the best bindings between the columns of the dataset and the graphical coordinates used by each visualization type.

To the best of our knowledge, though some tools (e.g., Spotfire and Tableau) integrate OLAP and analytics capabilities in the same environment, none of them allows users to formulate queries at a higher level of abstraction than OLAP (as done in the IAM using intentions), nor they support the automated *out-of-the-box* enrichment of cubes with insights obtained by analytics (as done in the IAM through enhanced cubes). For instance, Tableau^8^ enables OLAP sessions through a drag-and-drop metaphor. First, the user selects the levels and measures in which she is interested. Then, Tableau provides a *single* visualization based on such levels and measures (no cardinality checks are performed against level domains). Finally, the user can *manually* add some models (e.g., linear regression) and statistics. Thus, in comparison to the describe operator and the IAM, Tableau does not provide a high-level syntax (i.e., users must explicitly pick levels, measures, and models), an interestingness measure, and multiple visualizations combined with interesting highlights.

As stated in the Introduction, this paper extends our previous work (Chédin et al. 2020) in different ways. Specifically:

- While Chédin et al. (2020) only considered linear hierarchies, here cube schemata are defined in more general terms, allowing branches in hierarchies.
- The new definition of interestingness we propose here is based on three different facets: surprise, novelty, and peculiarity, while the one previously proposed considers peculiarity only.
- The definition of proxies we give here also covers situations where an intention changes both the group-by set and the selection predicate of the previous intention, and when there is no roll-up/drill-down relationship between the two group-by sets.
- The syntax of the describe operator has been extended by supporting multiple levels in the by clause and by allowing users to specify different sizes for each model.
- The visualization of enhanced cubes uses two more chart types to give users a more comprehensive and flexible description of data.
- The approach is evaluated through a comprehensive set of tests not only in terms of efficiency, but also of scalability, effectiveness, and formulation complexity.

## Conclusion

In this paper we have given a proof-of-concept for the IAM vision by delivering an end-to-end implementation of the describe operator, based on a novel measure of interestingness and relying on a visual metaphor to display enhanced cubes. This new measure of interestingness has been shown to be more effective than the one proposed by Chédin et al. (2020) in providing diversified highlights over enhanced cubes. We have also showed that our approach diminishes the effort for formulating complex analyses while ensuring that performances are compatible with near-real-time requirements of interactive sessions.

The main directions for future research we wish to pursue are: (i) evaluate the effectiveness of the approach by conducting extensive experiments with real users; (ii) optimize the computation of interestingness, especially for long sessions; and (iii) extend the approach to operate with *dashboards* of enhanced cubes.

## Footnotes

1 A *lattice* is a partially ordered set in which every two elements have a unique least upper bound and a unique greatest lower bound.

2 The outlierness (i.e., the anomaly score) of an observation in an isolation tree is the depth of the leaf containing this observation, i.e. the number of splittings required to isolate the observation.

3 Note that Marcel et al. (2019) describe one more facet of interestingness, namely, relevance. Relevance is not considered in this work because it would largely overlap with peculiarity.

4 As already mentioned, when no level is specified in the by clause for hierarchy *h*, it is implicitly assumed by*A**L**L*_*h*_

5 We deliberately chose not to differentiate the types of characters (e.g., keywords and free text) in the statements. Indeed, given the light syntax of the describe statements, weighing free text differently would favor describe over more verbose languages like SQL and Python.

6 www.ecdc.europa.eu/en/geographical-distribution-2019-ncov-cases

7 github.com/julianhyde/foodmart-data-mysql

8 https://www.tableau.com/
